# Effectiveness of stabilization methods for the immediate and short-term preservation of bovine fecal and upper respiratory tract genomic DNA

**DOI:** 10.1371/journal.pone.0300285

**Published:** 2024-04-02

**Authors:** Lee J. Pinnell, Cory A. Wolfe, Jake Castle, William B. Crosby, Enrique Doster, Paul S. Morley

**Affiliations:** 1 Veterinary Education, Research and Outreach Program, Texas A&M University, Canyon, TX, United States of America; 2 Department of Pathobiology and Population Medicine, College of Veterinary Medicine, Mississippi State University, Starkville, Mississippi State, United States of America; University of Illinois Urbana-Champaign, UNITED STATES

## Abstract

Previous research on stabilization methods for microbiome investigations has largely focused on human fecal samples. There are a few studies using feces from other species, but no published studies investigating preservation of samples collected from cattle. Given that microbial taxa are differentially impacted during storage it is warranted to study impacts of preservation methods on microbial communities found in samples outside of human fecal samples. Here we tested methods of preserving bovine fecal respiratory specimens for up to 2 weeks at four temperatures (room temperature, 4°C, -20°C, and -80°C) by comparing microbial diversity and community composition to samples extracted immediately after collection. Importantly, fecal specimens preserved and analyzed were technical replicates, providing a look at the effects of preservation method in the absence of biological variation. We found that preservation with the OMNIgene^®^•GUT kit resulted in community structure most like that of fresh samples extracted immediately, even when stored at room temperature (~20°C). Samples that were flash-frozen without added preservation solution were the next most representative of original communities, while samples preserved with ethanol were the least representative. These results contradict previous reports that ethanol is effective in preserving fecal communities and suggest for studies investigating cattle either flash-freezing of samples without preservative or preservation with OMNIgene^®^•GUT will yield more representative microbial communities.

## Introduction

Host-associated microbial communities play crucial roles in human health and the health of all animals [1–7]. Studies commonly target microbial communities of the gastrointestinal tract (GIT) where a large proportion of host-associated microbes reside, and GIT microbial communities have been implicated in a wide variety of health issues [8–18]. To this end, fecal samples are frequently collected and analyzed as a proxy for lower intestine microbial communities. Analogous to the past decade’s boom of microbiome research in people (i.e., Human Microbiome Project, American Gut Project), research investigating the role that microbial communities play in the health of livestock and agricultural environments has surged in recent years [5,19–24]. This is encouraged by a shifting dogma in animal sciences and veterinary medicine from culture-based microbiology studies of the past to culture-independent methods investigating features of interest (i.e., antimicrobial resistance, virulence, etc.) at the microbial community level using metagenomics or amplicon sequencing following the success of the human-associated microbiome research.

A large proportion of livestock-related microbiome studies target GIT or respiratory communities in cattle and other species. The bovine GIT microbiome has been linked to several diseases that are important both clinically and financially, and is associated with growth, production, and efficiency of cattle [19,20,25]. The composition of microbial communities in the respiratory tract of cattle has increasingly been implicated in pneumonia or bovine respiratory disease (BRD), which is the most common disease requiring antimicrobial drug treatment among beef cattle in North America [5,21,26–28]. Researchers commonly collect fecal samples to characterize GIT communities and use swabs to collect upper respiratory tract secretion samples. Ideally, samples would be immediately processed or frozen at ultralow temperatures to minimize changes in microbial community compositions. However, when samples are collected away from research facilities this is not always convenient or possible. For example, our research group has conducted research in which human volunteers collected feces from themselves or their animals over time. Additionally, research frequently occurs at agriculture facilities that are hundreds and even thousands of miles away from the researchers’ home laboratories with ultracold storage capability. Thus, storage conditions on site and during transportation to the laboratory could create significant biases in results related to microbial ecology. Different preservative methods have been proposed for stabilizing microbial community structures, especially for investigation of human fecal microbiota [29–32]. However, only a few published studies have investigated preservation methods using animal feces [33–35], and notably, these investigations did not include fecal samples from cattle. Additionally, little published research is available regarding impacts of preservatives on other important sample types such as respiratory secretions. Cost is another important consideration for large microbiome studies, and costs for preservation methods that have been advocated can vary from a few cents (e.g., ethanol) to several dollars (e.g., commercially available solutions) per sample.

Significant systematic differences or biases in microbiome data that are associated with different sample preservation methods have been clearly demonstrated in studies of human feces [36], even though changes in community composition are often smaller than differences between sample types or among study subjects [35]. Regardless, small systematic differences in non-dominant taxa can have far reaching consequences for animal and ecosystem health [5]. Therefore, there is a need to expand our understanding of how stabilization methods shift microbial community composition in the absence of variation associated with differing sample types or study subjects (i.e., biological variation) to isolate the effect of different stabilization methods.

This study used very deep 16S rRNA gene sequencing (~ 1 million sequence reads per sample) to A) systematically compare sample preservation factors (preservative solution, temperature, and time) that might be affect to short- to intermediate-term intervals between collection of cattle fecal samples and receipt at a distant laboratory; and B) investigate impacts of using ethanol as an inexpensive preservative of bovine respiratory secretion samples.

## Methods

### Sampling and experimental design

**Fig 1:**
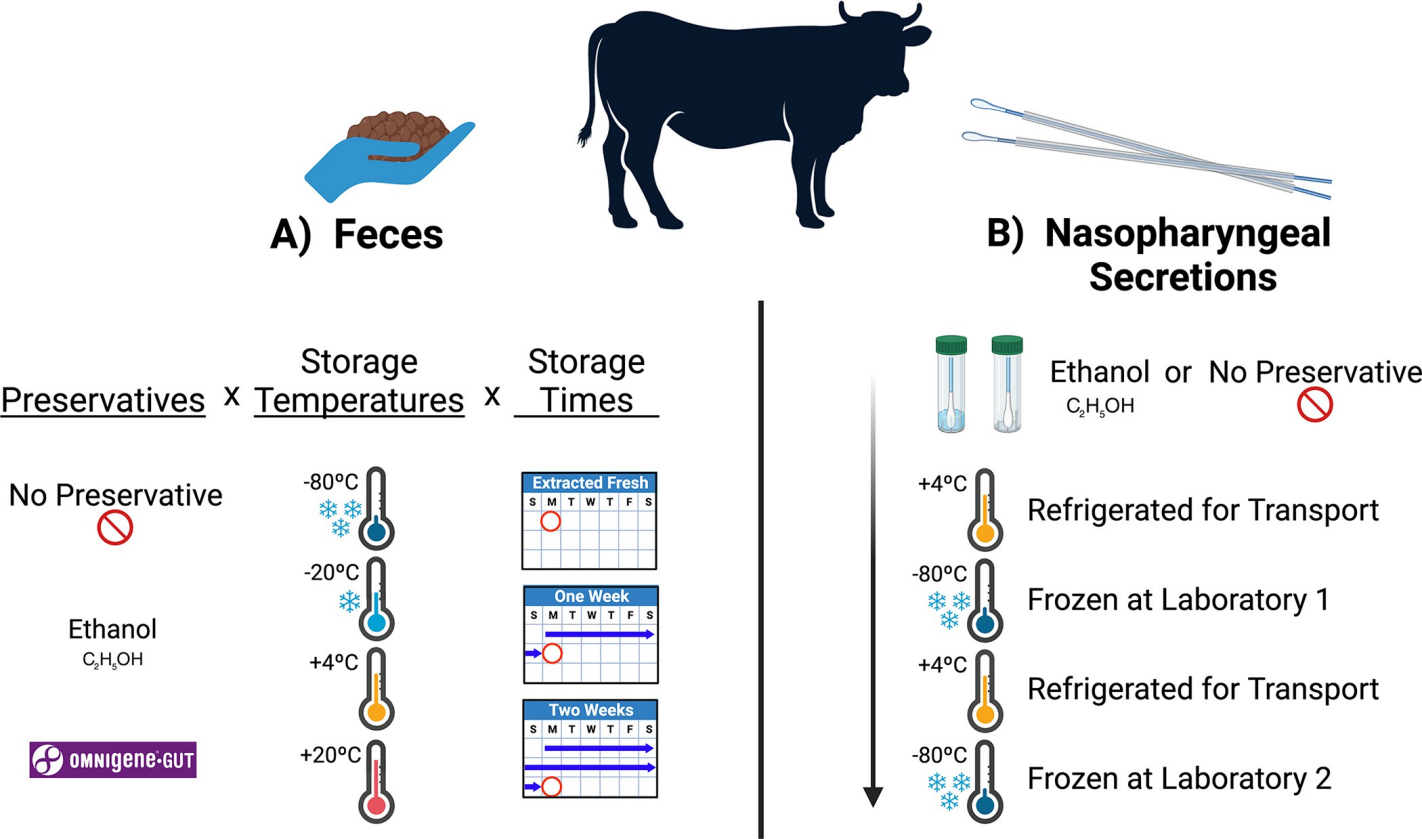
Schematic of sample processing strategy for parts A and B of this study. Created with BioRender.com.

Part A) A single bovine fecal sample was collected per rectum using a sterile disposable palpation sleeve, place into a sterile container, and delivered to the laboratory where it was aliquoted into one of three preservative treatment groups (Fig 1): no treatment, OMNIgene^®^•GUT (DNA Genotek^®^, Stittsville, Ontario, Canada) stabilizing solution (500mg feces /2mL of solution), or 100% molecular grade ethanol (500mg feces /2mL of EtOH). Tubes were shaken and vortexed to homogenize the feces and preservative solutions. P1000 pipette tips were aseptically cut to widen the tip orifice enough to allow 250μL of homogenous sample-mixture to be pipetted into 2ml tubes (approximately 60mg of stool sample per aliquot). This was repeated 36 times for both treatment groups, OMNIgene and ethanol. 50mg of stool was then weighed into 36 more empty 2mL tubes for the non-treatment group, totaling 108 samples across all three experimental groups. Four aliquots from each preservative treatment group were immediately processed for DNA isolation (day 0) and the remaining 2mL tubes were stored at four temperatures (-80°C, -20°C, 4°C, or ambient room temperature ~20°C) for either 7 or 14 days before DNA isolation was performed. These time points were selected to represent intervals that might be used when storing samples onsite at production facilities before transporting them to a laboratory for further processing.

Part B) Commercially available, sterile, double-guarded, cotton fiber swabs (29.5 in / 74.9 cm; E9-5200, Continental Plastic, Delavan, WI) were used to obtain samples of respiratory secretions from the deep nasopharynx of feedlot cattle as previously described [21]. The 16 cattle enrolled in Part B of this study were sampled on day 14 after arrival at the West Texas A&M University Research Feedlot on May 14, 2020. Briefly, the external nares were cleaned with a paper towel to remove superficial secretions and dirt, and 2 nasopharyngeal (NP) swabs were passed sequentially through the right nostril to collect respiratory secretions from the distal nasopharynx. The swab tip from one sample was aseptically cut and placed into a sterile 5mL tube containing 0.5ml of 100% molecular grade ethanol, and the other was swab tip was placed in a sterile tube without preservative. All samples were kept on ice and transported to Laboratory 1, where they were placed in an ultracold (-80°C) freezer within 6 hours of collection. Two weeks later, all samples were placed in a styrofoam lined box with standard ice packs and shipped by overnight courier to Laboratory 2. They were received at Laboratory 2 approximately 24 hours after removal from the ultracold freezer at Laboratory 1, then frozen again at -80°C until processed for DNA isolation 4 weeks later. Storage and transportation used in Part B were intended to represent a scheme that might be used when collecting samples onsite at a production facility before transporting them to a laboratory for further processing.

### DNA isolation, 16S rRNA library preparation and sequencing

DNA isolation of both fecal (50-70mg starting material) and nasal swab samples (1 entire swab tip) was performed using the QIAmp PowerFecal DNA kit (Qiagen, Hilden Germany) according to manufacturer’s instruction with slight modifications to the cell lysis step. Samples were heated at 65°C for 10-minutes, then homogenized using 5 cycles of bead beating (1-minute pulses with a 5-minute rest between each pulse). The elution buffer was also heated to 65°C before being pipetted directly onto the silica membrane and left for 5 minutes to allow for complete rehydration. Following isolation, DNA was quantified (ng μL^-1^) using a Qubit flex fluorometer (Thermo Fisher Scientific, Waltham, MA, USA).

The V3-V4 region of the 16S rRNA gene was amplified using the 341F (5’– CCTACGGGNGGCWGCAG– 3’) and 785R (5’– GACTACHVGGGTATCTAATCC– 3’) primer pair (Integrated DNA Technologies, Inc, Coralville, IA). Preparation of amplicon libraries for 16S rRNA gene sequencing was conducted according to Illumina’s protocol [37]. The resulting pooled amplicon library was sequenced on an Illumina NovaSeq instrument using paired-end chemistry (2 x 250bp) at the University of Colorado Anschutz Medical Campus’ Genomics and Microarray Core. As negative controls, equal volumes of nuclease-free sterile water were included with each batch of samples processed. No sequence reads passed the quality filtering steps of DADA2 (described below) from the negative controls, and therefore they were not included in further downstream analysis. A ZymoBIOMICS Microbial Community DNA Standard (Zymo Research, Irvine, CA, USA) was included as a positive control for PCR and to check for bias introduced during library preparation and sequencing. The number of reads per fecal sample ranged from 541,616 to 3,495,132 with a mean sequence depth of 951,895. The number of reads per respiratory swab sample ranged from 343,811 to 10,615,904 with a mean sequence depth of 3,045,236 reads per sample. All sequence reads were made available through BioProject PRJNA1043101 at the NCBI’s Sequence Read Archive.

#### Bioinformatics

Demultiplexed paired-end reads were imported into QIIME2 version 2020.11 [38] and DADA2 [39] was used to filter reads for quality, remove chimeric sequences, merge overlapping paired-end reads, and generate amplicon sequence variants (ASVs). Forward and reverse reads were trimmed at 17 bp and 21 bp and truncated at 248 bp and 250 bp, respectively. Taxonomy was assigned using a Naïve Bayes classifier trained on the SILVA 138 99% OTUs database, where sequences had been trimmed to include only the base pairs from the V3-V4 region bound by the 341f/785r primer pair. Reads that mapped to chloroplast and mitochondrial sequences were filtered from the representative sequences and ASV table using the ‘filter_taxa’ function, and a midpoint-rooted phylogenetic tree was then generated using the ‘q2-phylogeny’ pipeline with default settings, which was used to calculate phylogeny-based diversity metrics. The proportion of ASVs classified at each taxonomic rank for fecal and swab samples can be found in S1 Table. Briefly, 93.7% of fecal ASVs were classified at the rank of genus, and >99% of fecal ASVs were classified at higher ranks (i.e., family, order, class, phylum). Similarly, 95.4% of respiratory ASVs were classified at the rank of genus, while > 99% of respiratory ASVs were classified at higher ranks (i.e., family, order, class, phylum).

Data and metadata were then imported into an R programming environment using the phyloseq package [40]. ASV counts for each sample were then normalized using cumulative sum scaling [41] and beta-diversity was analyzed using weighted, generalized, and unweighted UniFrac distances [42,43]. From these distances, principal co-ordinates analysis (PCoA) was performed and plotted, and a permutational multivariate analysis of variance (PERMANOVA) was used to test for significant differences in community structure using the vegan [44] and pairwiseAdonis [45] packages. To ensure significant differences were not the result of unequal dispersions of variability, permutational analyses of dispersion (PERMDISP) were conducted for all significant PERMANOVA outcomes using the vegan package. Hierarchal clustering was performed using Ward’s agglomeration clustering method [46] on generalized UniFrac distances and the ‘hclust’ function. Further, the relative abundances of ASVs within each sample were calculated and plotted using phyloseq.

#### Statistical analyses

Unless otherwise stated, R version 4.2.1 [47] was used for statistical analysis of data. Pairwise Wilcoxon rank-sum analyses of variance were performed using a Benjamini-Hochberg correction for multiple comparisons. Differences in beta diversity were tested using pairwise PERMANOVA with a Benjamini-Hochberg correction for multiple comparisons and 9,999 permutations. Additionally, pairwise PERMDISPs were carried out for all significant PERMANOVA outcomes using 9,999 permutations to test for differences in the variability of dispersions. Correlation between the relative abundances of ASVs was tested using Pearson’s correlation co-efficient.

#### Regulatory approval

The protocol used to collect the fecal sample used in this project was reviewed and approved by the Colorado State University Research Integrity and Compliance Review Office and determined to be exempt from Institutional Animal Care and Use Committee oversight. Protocols used in collection of respiratory swabs for this research were reviewed and approved by the West Texas A&M University Institutional Animal Care and Use Committee (Protocol# 2020.04.003).

## Results

**Fig 2:**
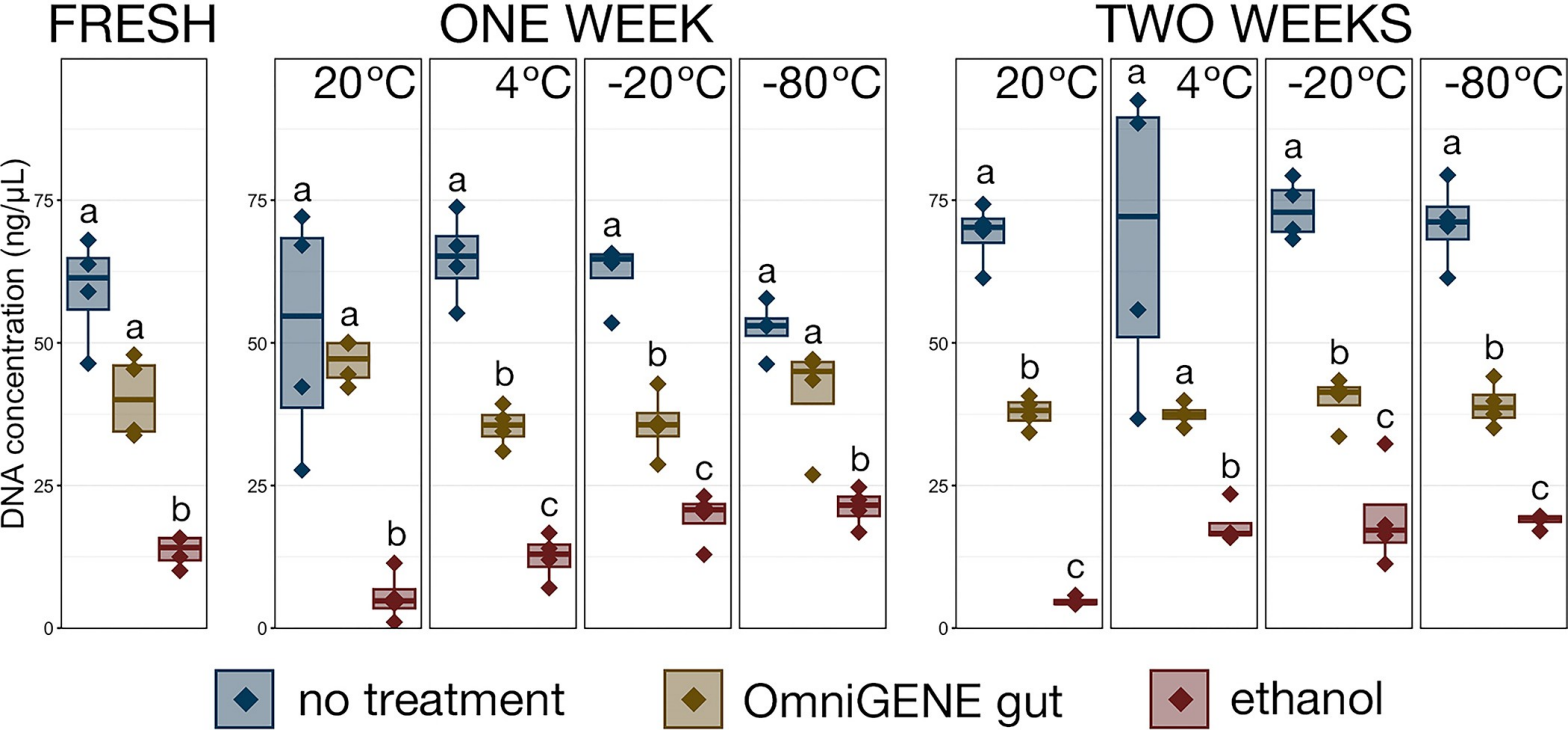
Boxplots demonstrating the concentration of DNA isolated from bovine fecal samples stored with each of the preservation methods (no treatment, OMNIgene^®^•GUT solution, ethanol) at each of the timepoints and each storage temperature. Significant differences between treatment methods at each timepoint and temperature are illustrated by different letters (pairwise Wilcoxon rank-sum with Benjamini-Hochberg, n = 3–4, p < 0.05).

*Part A) Impact of stabilization solutions on DNA yields following isolation*. We compared the effect that stabilization solutions had on the yield of DNA recovered from samples without stabilization solution and those stabilized with OMNIgene^®^•GUT and ethanol (Fig 2 and S2 Table). In general, stabilization with either buffer resulted in significantly lower DNA yields than untreated samples, but samples stabilized in ethanol had substantially lower DNA yield across all time points when compared with untreated samples and those preserved with OMNIgene^®^•GUT (Fig 2 and S2 Table; pairwise Wilcoxon rank-sum with Benjamini-Hochberg correction; n = 3–4, p < 0.05). In samples extracted immediately after sample collection, DNA yields from ethanol stabilized samples were over 4-fold lower than samples without treatment and 3-fold lower than OMNIgene stabilized samples (Fig 2 and S2 Table no treatment 59.30 ng·μL^-1^ ± 4.68 SEM, OMNIgene®•GUT 40.45 ng·μL^-1^ ± 1.39 SEM, ethanol 13.54 ng·μL^-1^ ± 3.62 SEM). There were no significant differences in DNA yield between the four storage temperatures within any of the treatment groups after 7-days or 14-days of preservation (Fig 2 and S2 Table; pairwise Wilcoxon rank-sum with Benjamini Hochberg, n = 3–4, p > 0.05), though an increased yield in ethanol stabilized samples that were frozen compared to those stored at 4°C and 20°C was notable (p = 0.06).

### Immediate effects of stabilization solutions on fecal microbial communities

**Fig 3:**
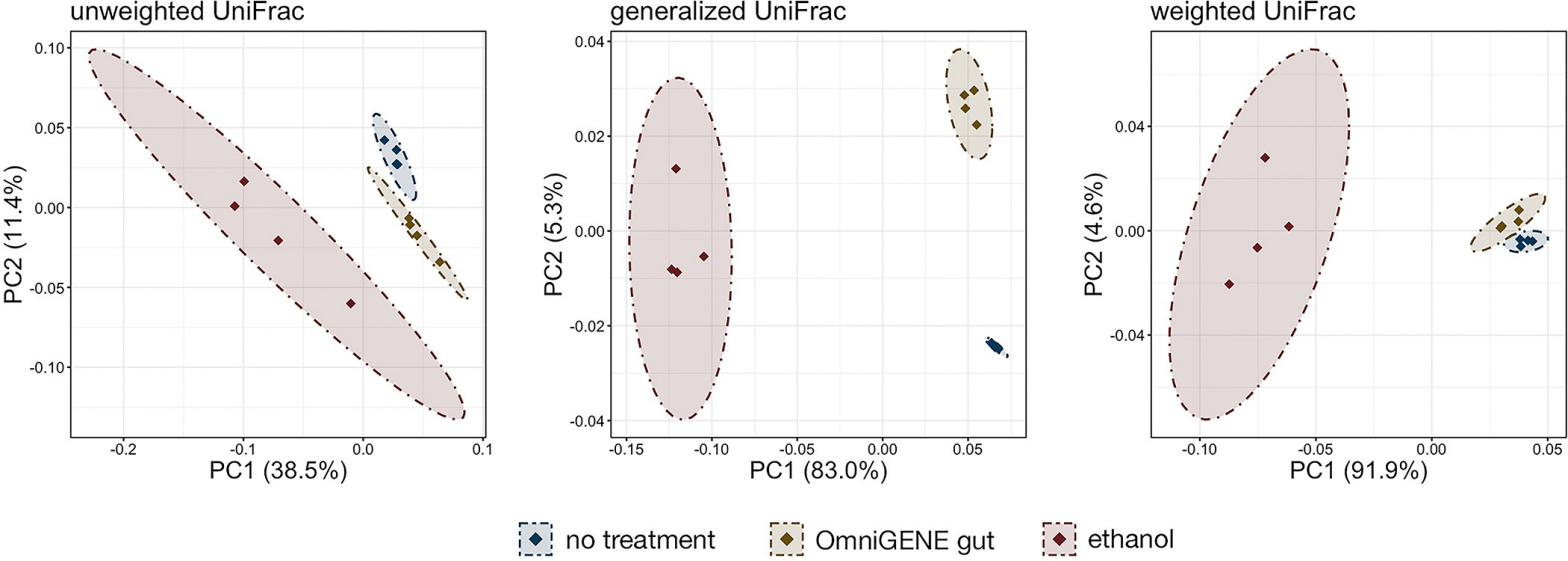
Principal co-ordinates analysis (PCoA) based on unweighted, generalized, and weighted UniFrac distances from samples extracted immediately after collection and stabilized with OMNIgene^®^•GUT solution, 100% ethanol, or given no stabilization solution (no treatment). Shaded areas and dashed lines represent 95% confidence ellipses.

**Fig 4:**
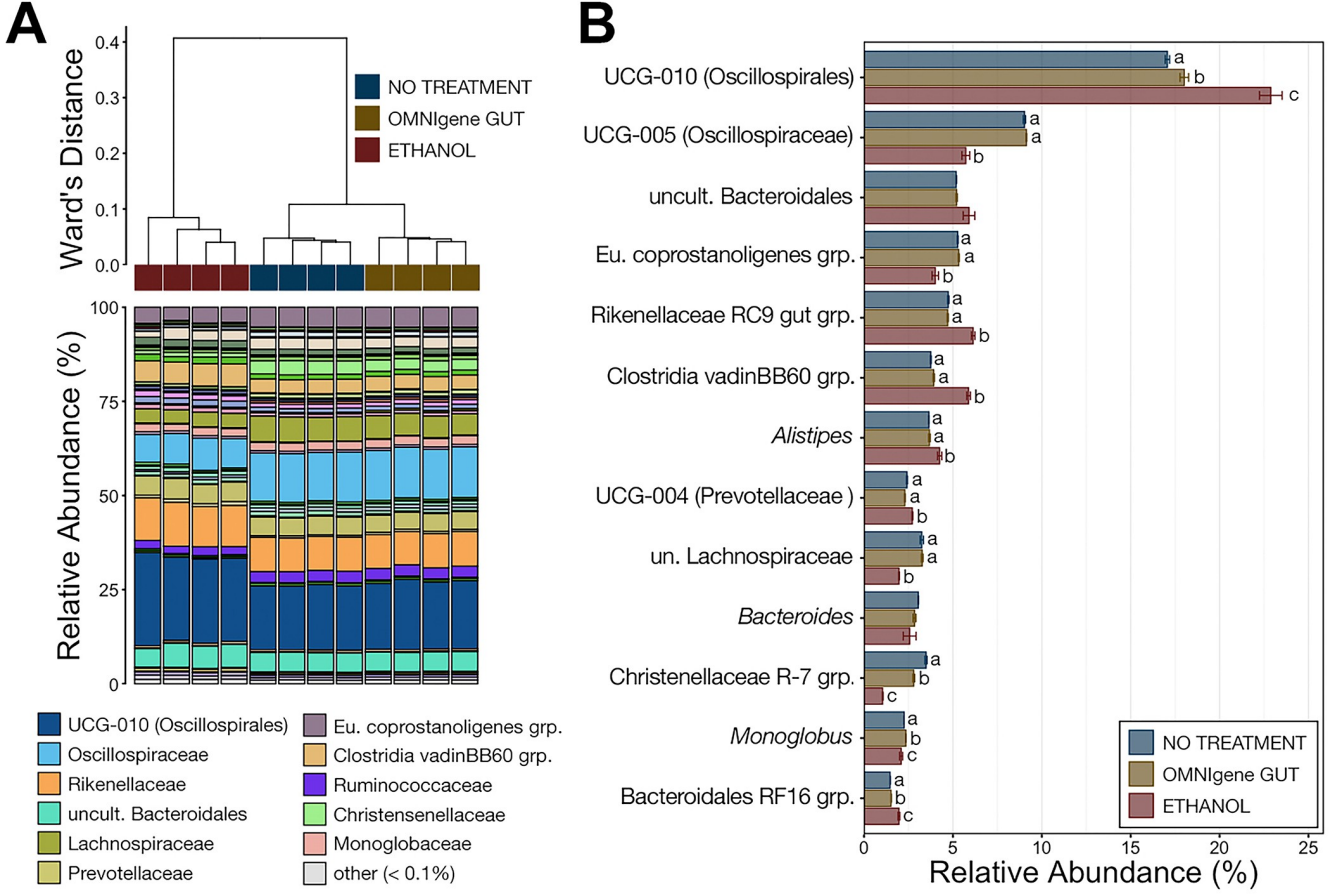
**A)** The relatedness of microbial communities from samples extracted immediately after collection and stabilized with OMNIgene^®^•GUT solution, 100% ethanol, or given no stabilization solution (no treatment). Hierarchal clustering was performed on generalized UniFrac distances using Ward’s agglomeration method. The bar plot illustrates the relative abundance of microbial families within each individual family. The ten most abundant families are displayed in the legend. **B)** Barplots demonstrating the relative abundance of the 13 genera comprising greater than 1% of the overall microbial community across all samples extracted immediately after collection. Error bars represent standard error of the mean and significant differences in the relative abundance of genus between treatments are illustrated by different letters (pairwise Wilcoxon rank-sum with Benjamini-Hochberg, n = 4, p < 0.05).

**Fig 5:**
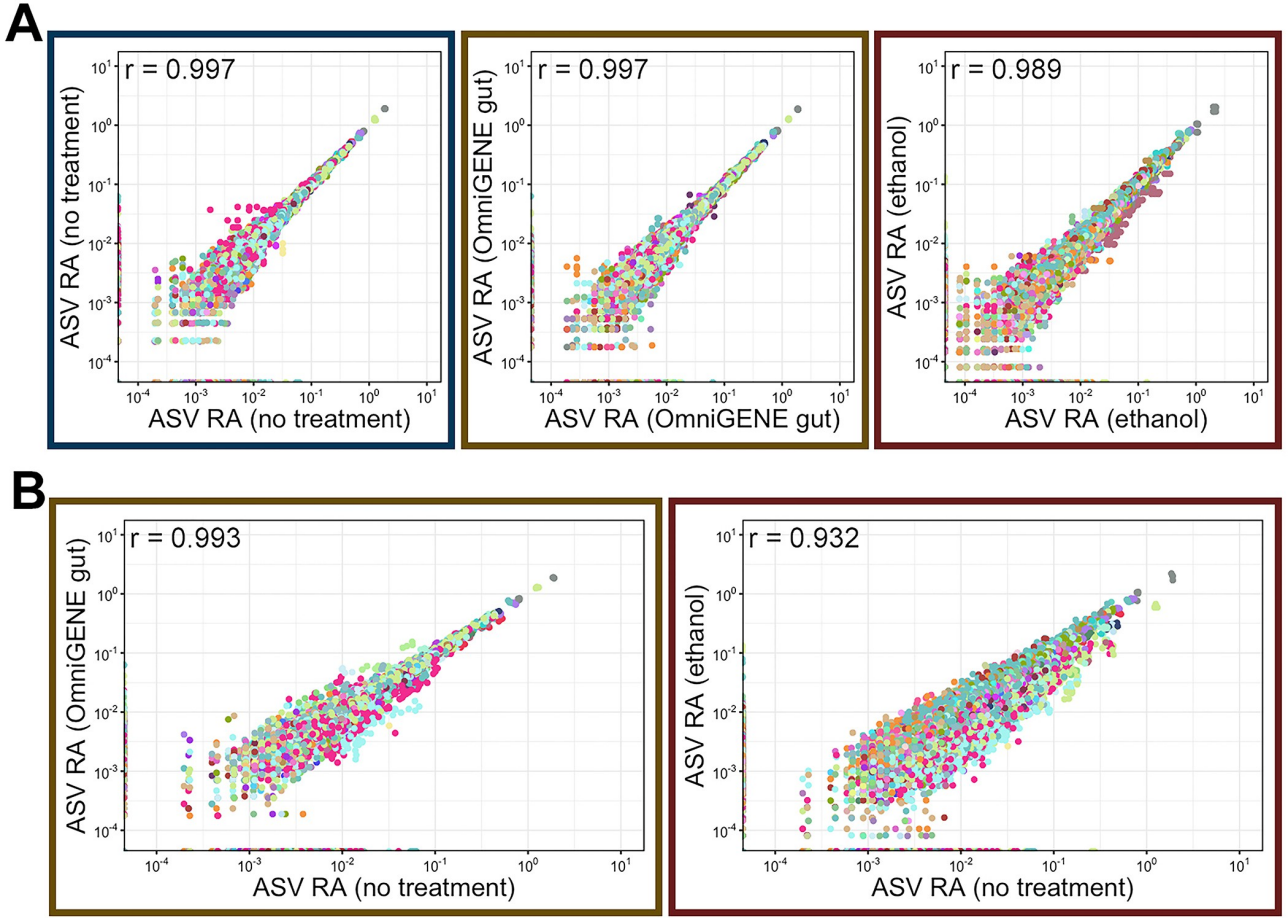
Scatterplots showing the correlation between the relative abundance of every ASV between **A)** the four replicates within each treatment group (OMNIgene^®^•GUT solution, 100% ethanol, no treatment) and **B)** each treatment and the no treatment group from samples extracted immediately after collection. ASVs are colored by taxonomic family, and the Pearson correlation coefficient is displayed in the top right corner of each panel.

To isolate the effect of the preservation method in the absence of storage time we compared microbial community diversity and composition from fresh samples extracted immediately after collection and adding preservative to the sample. A principal-coordinate analysis (PCoA) based on unweighted, generalized, and weighted UniFrac values revealed that in the absence of other factors, addition of a stabilization solution was associated with subtle yet statistically significant microbial community composition shifts (Fig 3; PERMANOVA with Benjamini-Hochberg correction, n = 4 per group, adj-p < 0.05). Microbial communities in samples without stabilization buffer were different from samples stabilized with OMNIgene^®^•GUT and ethanol, but those stabilized with ethanol showed the greatest differences. Untreated and ethanol stabilized samples are clearly separated along PC1 (Fig 3) and ethanol stabilized samples formed their own clade that was most distantly related (Fig 4A). This is further exemplified by the fact that preservation method explained far more of the variation in community structure for ethanol stabilized samples versus OMNIgene^®^•GUT stabilized samples (S3 Table; 88% ethanol vs. no treatment and 49% OMNIgene^®^•GUT vs. no treatment based on generalized UniFrac distances). The difference in ethanol stabilized samples was largely explained by changes in the relative abundances of major lineages. For example, the relative abundances of the two most abundant families (Oscillospirales UCG-010 & Oscillospiraceae) and the genera comprising those families were significantly different between ethanol stabilized communities versus those receiving no treatment (Fig 4A and 4B; pairwise Wilcoxon rank-sum with Benjamini-Hochberg correction, p < 0.05). Further, the relative abundance of 11 of the 13 genera comprising greater than 1.0% of the overall microbial community across all fresh samples was significantly different in ethanol stabilized versus untreated fecal samples, while only three differed in OMNIgene^®^•GUT stabilized samples (Fig 4B; pairwise Wilcoxon rank-sum and Benjamini-Hochberg correction, n = 4, p < 0.05). Samples stabilized with ethanol also exhibited wider variance amongst themselves at the ASV level (correlation coefficient = 0.989), while those stabilized with OMNIgene^®^•GUT had similar inter-sample variance amongst themselves to samples receiving no stabilization solution (Fig 5A; OMNIgene^®^•GUT r = 0.997, no treatment r = 0.997). Additionally, when compared to untreated samples at the ASV level, samples stabilized with ethanol were less similar (Fig 5B; r = 0.932) than those stabilized with OMNIgene^®^•GUT (Fig 5B; r = 0.993).

### Changes in bovine fecal microbial community composition following short-term storage at different temperatures

**Fig 6:**
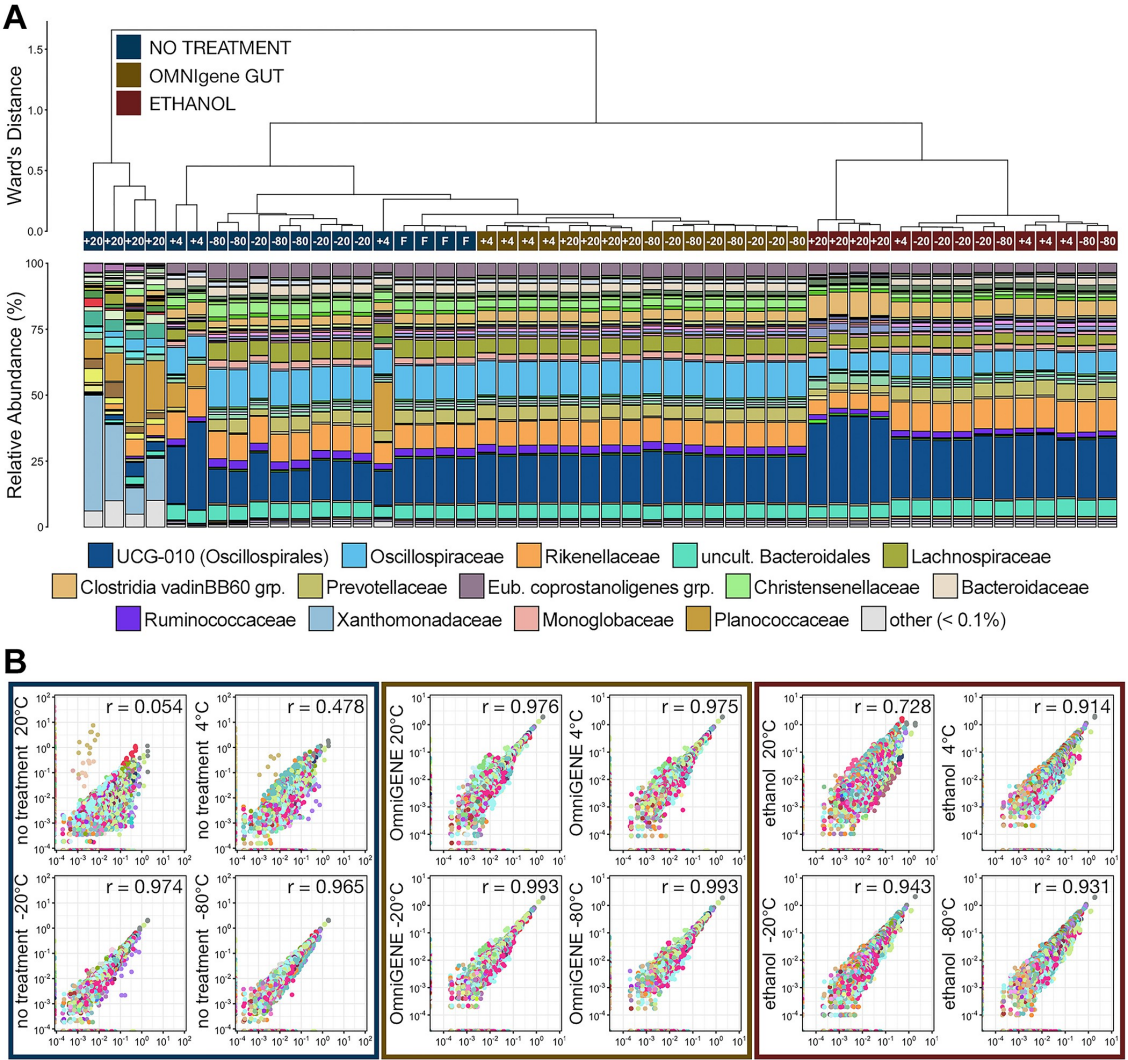
**A)** The relatedness of microbial communities from samples extracted after 7-days storage at room temperature, 4°C, -20°C, or -80°C and stabilized with OMNIgene^®^•GUT solution, 100% ethanol, or given no stabilization solution (no treatment). Hierarchal clustering was performed on generalized UniFrac distances using Ward’s agglomeration method. The bar plot illustrates the relative abundance of microbial families within each individual family. The fifteen most abundant families across all 7-day storage samples are displayed in the legend. **B)** Scatterplots showing the correlation between the relative abundance of every ASV between samples from each treatment group and storage temperature after 7-days storage and no treatment samples that were extracted immediately.

Following 7-days of storage, communities stabilized with OMNIgene^®^•GUT and stored at any temperature–including room temperature–were the most similar to fresh communities and formed their own clade (Fig 6A). Within the OMNIgene clade, samples stored below freezing (i.e., -20°C, -80°C) clustered together and samples stored at 4°C and room temperature clustered together (Fig 6A). Samples stabilized with OMNIgene^®^•GUT and stored at -20°C (r = 0.993) and -80°C (r = 0.993) had the smallest shifts in the relative abundances across ASVs versus fresh samples following 7 days storage (Fig 6B). OMNIgene^®^•GUT stabilized samples that were stored at 4°C (r = 0.975) and 20°C (r = 0.976) exhibited a slightly larger difference in microbial community composition, which was like those observed among samples frozen without stabilization solution (Fig 6B).

Community composition in untreated control samples that were stored below freezing (i.e., -20°C, -80°C) for 7 days were the next most similar to fresh communities (Fig 6A). Those stored at -20°C, -80°C, and 4°C formed their own clade and were the next most similar to fresh communities, while those stored at room temperature were by far the most different (Fig 6A). Interestingly, despite more noticeable differences at the family level in untreated samples stored at 4°C for 7 days, the microbial communities, as a whole, were more similar to fresh samples then those stored in ethanol at any temperature. Members of the families Planococcaceae and Xanthomonadaceae, which comprised less than 0.05% of the overall communities in all stabilized (i.e., OMNIgene^®^•GUT and ethanol) communities and fresh untreated communities, were significantly more predominant within communities from untreated samples stored at 4°C and room temperature for 7 days (Fig 6A; pairwise Wilcoxon rank-sum test with Benjamini-Hochberg correction, n = 3–4, p < 0.05). There was a considerable difference between the taxonomic relative abundance in untreated communities stored above and below freezing; proportions of different ASVs in frozen samples were similar to communities found in fresh samples (-80°C r = 0.965; -20°C r = 0.974) while those of samples stored at 4°C (r = 0.478) and room temperature (r = 0.054) were vastly different (Fig 6B).

Microbial communities in fecal samples stabilized with ethanol and stored for 7 days at all temperatures formed their own clade; other than microbial communities of untreated control samples stored at room temperature, communities of samples treated with ethanol and stored for 7 days were the most different from those of fresh untreated samples (Fig 6A). Within the ethanol stabilized clade, communities from samples stored at room temperature were considerably different than communities stored a cooler temperature. This was largely a result of relative abundance increases in Oscillospirales UCG-010 and the Clostridia vadinBB60 group and decreases in Oscillospiraceae, Rikenallaceae, and Prevotellaceae (Fig 4A). While ethanol stabilized communities were more distantly related to fresh communities than untreated communities stored at 4°C, it is evident that the proportions of ASVs in ethanol stabilized communities were more similar to communities in fresh untreated samples (Fig 6B). However, the proportions of ASVs from communities stabilized in ethanol and stored at -20°C (r = 0.943) and -80°C (r = 0.931) exhibit larger shifts than those not stabilized and stored at the same temperatures (Fig 6B). After 7 days, the proportions of ASVs within communities stored in ethanol at 4°C (r = 0.914) demonstrated much smaller shifts than those not stabilized (Fig 6B).

**Fig 7:**
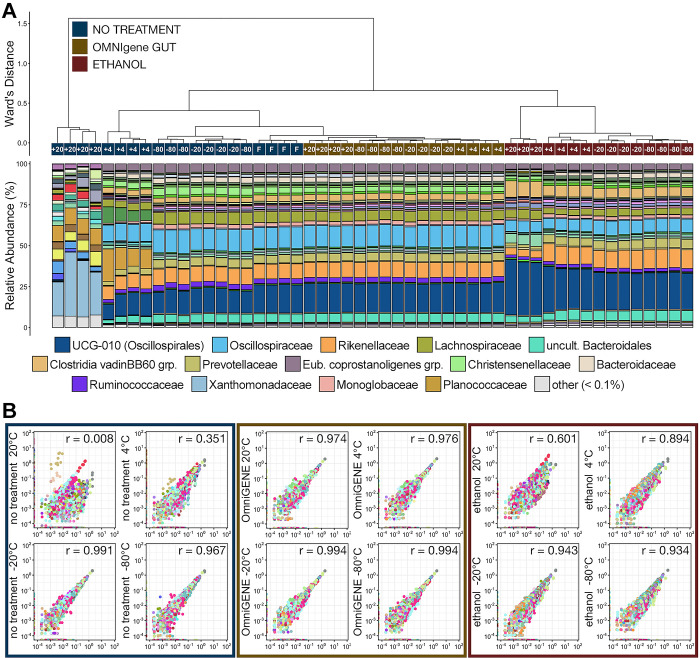
**A)** The relatedness of microbial communities from samples extracted after 14-days storage at room temperature, 4°C, -20°C, or -80°C and stabilized with OMNIgene^®^•GUT solution, 100% ethanol, or given no stabilization solution (no treatment). Hierarchal clustering was performed on generalized UniFrac distances using Ward’s agglomeration method. The bar plot illustrates the relative abundance of microbial families within each individual family. The fifteen most abundant families across all 14-day storage samples are displayed in the legend. **B)** Scatterplots showing the correlation between the relative abundance of every ASV between samples from each treatment group and storage temperature after 14-days storage and no treatment samples that were extracted immediately.

The relationship between the similarities of communities preserved via different stabilization methods (i.e., none, OMNIgene^®^•GUT, ethanol) and different temperatures (i.e., -80°C, -20°C, 4°C, room temperature ~20°C) remained the same following 14-days storage (Fig 7A). Like after 7-days storage, microbial communities stabilized with OMNIgene and stored at any temperature were the most similar to communities of fresh untreated fecal samples and once again formed clades largely based on storage temperature (Fig 7A). Samples stabilized with OMNIgene and stored below freezing (-20°C r = 0.994; -80°C r = 0.994) once again had the smallest shifts in ASV relative abundance versus fresh samples after 14 days storage (Fig 7B). OMNIgene^®^•GUT stabilized samples that were stored at 4°C (r = 0.976) and 20°C (r = 0.974) exhibited a slightly larger shift in ASV relative abundance which were similar to those observed in untreated samples and stored at -20°C and -80°C. All four storage temperatures for OMNIgene^®^•GUT stabilized samples exhibited nearly identical shifts in ASV proportions at 14-days to those seen after 7-days (Figs 6B and 7B, respectively).

As was the case at 7-days of storage, communities stored without a stabilization solution and frozen at -20°C or -80°C were the next most similar to fresh communities (Fig 7A) and exhibited shifts in ASV relative abundance similar to those of OMNIgene stabilized communities (Fig 7B). The differences observed at 7-days in untreated communities stored at 4°C and room temperature were exacerbated, with significantly increased relative abundances of Planococcaceae and Xanthomonadaceae within communities stored at 4°C and room temperature, respectively (Fig 7A; pairwise Wilcoxon rank-sum test with Benjamini-Hochberg correction, n = 4, p < 0.05). Large shifts in the relative abundances of ASVs within these samples was once again observed (4°C r = 0.351; RT r = 0.008), and these shifts had also increased when compared to 7-days storage (Figs 6B and 7B).

Communities stabilized with ethanol and stored for 14-days again formed their own clade that, except for untreated communities stored at room temperature, was the least similar to fresh samples (Fig 7B). Samples stored below freezing for 14 days exhibited similar shifts (-20°C r = 0.943; -80°C r = 0.931) in ASV relative abundances as those stored for 7-days, but these shifts were greater than all OMNIgene stabilized communities and untreated communities stored below freezing (Fig 7B). At 4°C and room temperature, ethanol stabilized communities (4°C r = 0.895; RT r = 0.602) had much smaller changes in ASV proportions compared to those in untreated communities (Fig 7B) following 14-days storage, but much larger changes compared to those stabilized with OMNIgene^®^•GUT. Additionally, shifts in ASV relative abundance increased between 7-days and 14-days storage (Figs 6B and 7B, respectively).

### Part B) DNA isolation yields and changes in bovine respiratory microbial community composition following stabilization with ethanol

**Fig 8:**
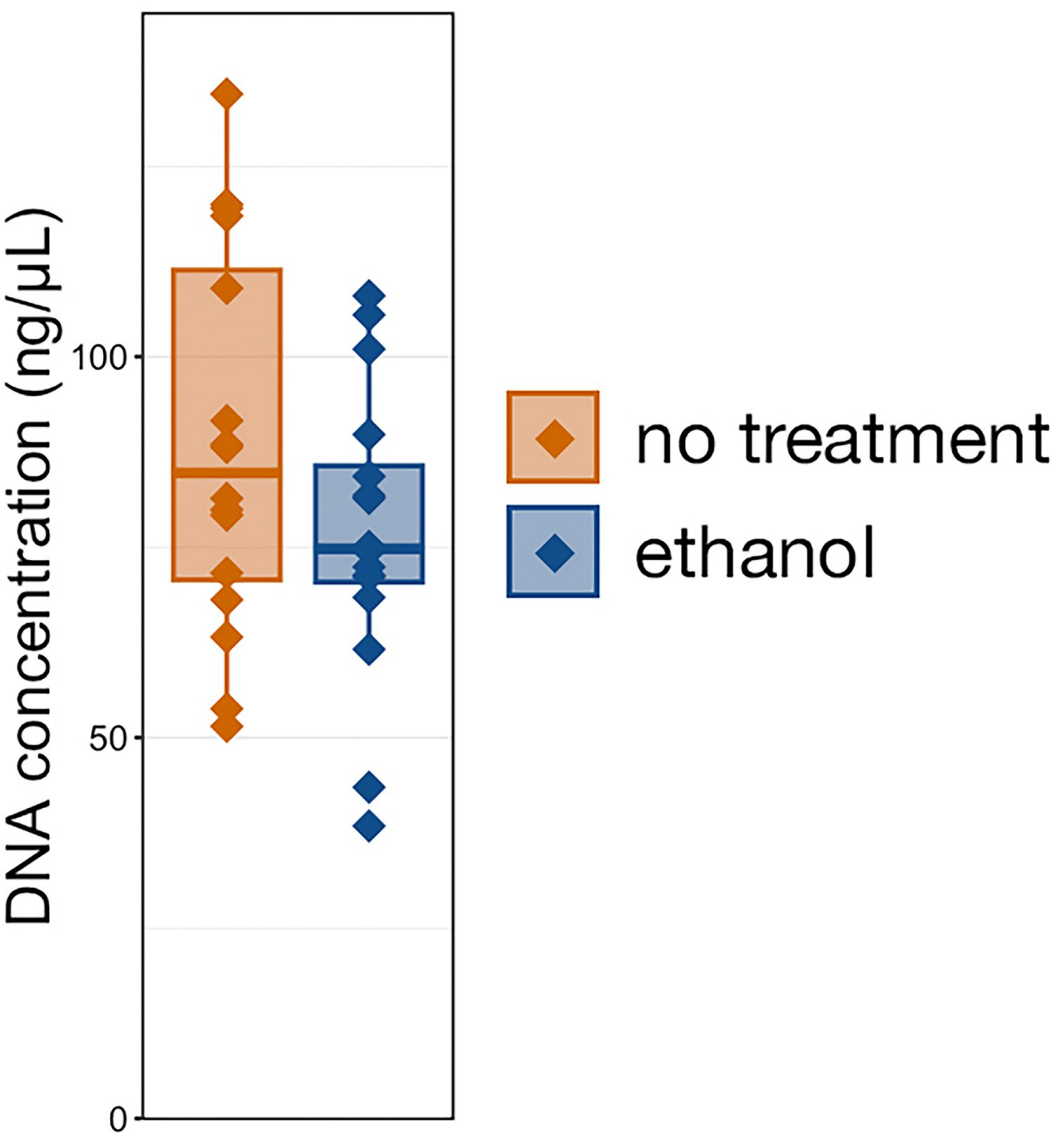
Boxplot demonstrating the concentration of DNA isolated from bovine respiratory samples stored with 100% ethanol or no stabilization solution. No significant differences between treatment methods were identified (pairwise Wilcoxon rank-sum with Benjamini-Hochberg, n = 16, p > 0.05).

Unlike with fecal samples, there was no significant difference in the DNA yield resulting from samples stabilized with ethanol versus untreated samples (Fig 8 and S4 Table; Kruskal-Wallis ANOVA, n = 16, p > 0.05). Though not significant, yields were slightly lower from ethanol stabilized samples than from untreated samples (no treatment 88.65 ng·μL^-1^ ± 6.30 SEM, ethanol 76.76 ng·μL^-1^ ± 4.84 SEM).

**Fig 9:**
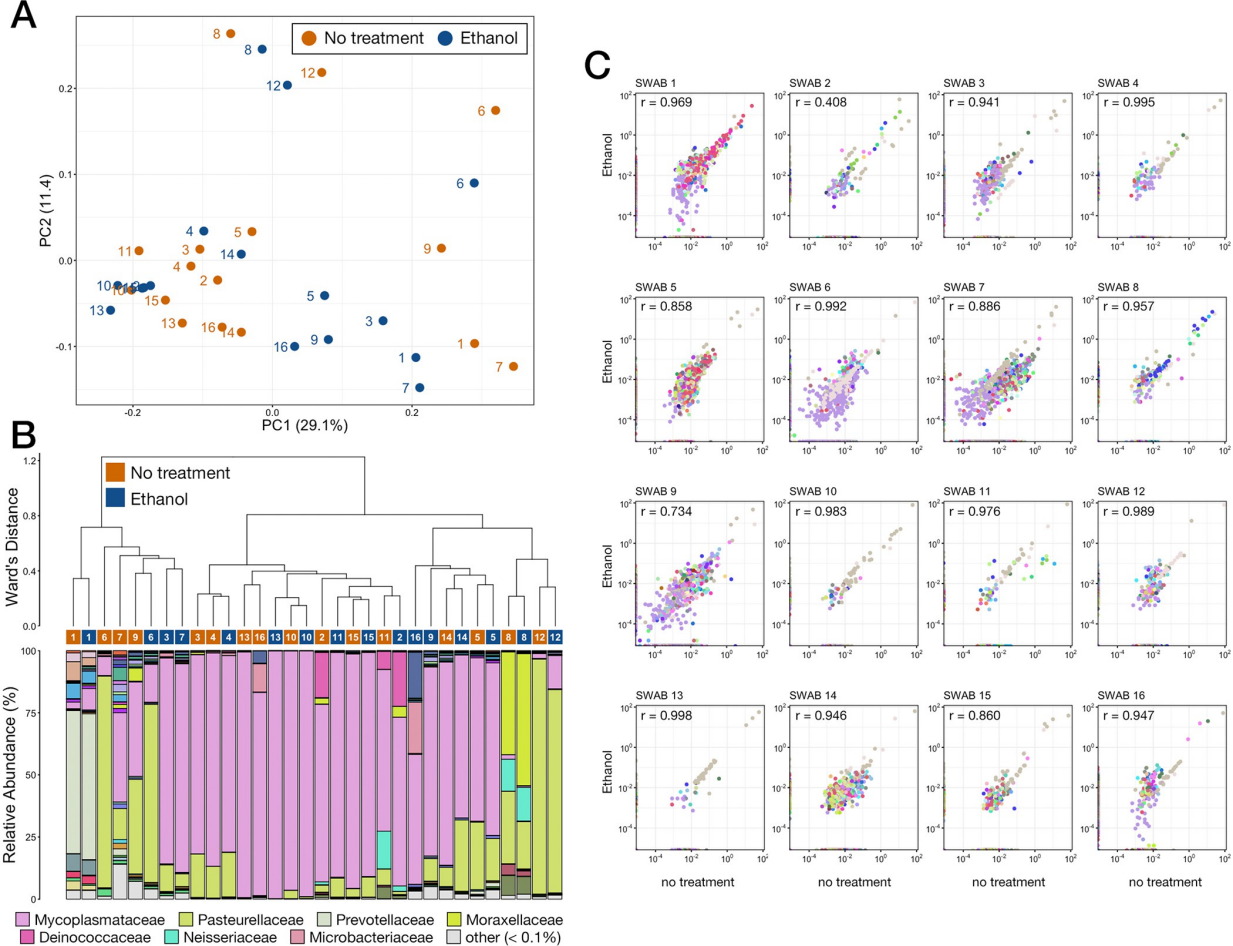
**A)** Principal co-ordinates analysis (PCoA) based on generalized UniFrac distances from respiratory samples stabilized with 100% ethanol or given no stabilization solution (no treatment). Numbers (1–16) represent the individual animal that both the stabilized and untreated samples were collected from. **B)** The relatedness of microbial communities from respiratory samples stabilized with 100% ethanol or given no stabilization solution (no treatment). Hierarchal clustering was performed on generalized UniFrac distances using Ward’s agglomeration method. The bar plot illustrates the relative abundance of microbial families within each individual family. The eight most abundant families across all respiratory samples are displayed in the legend. **C)** Scatterplots showing the correlation between the relative abundance of every ASV between samples stabilized with 100% and those that did not receive stabilization solution for each of the 16 animals sampled.

To investigate the effects of ethanol stabilization on bovine respiratory microbial communities we compared the microbial community composition in treated samples to those of untreated swab samples collected at the same time. A PCoA based on generalized UniFrac values illustrated that samples did not cluster based on preservation method and that, in general, ethanol stabilized communities tended to cluster with their untreated counterpart (Fig 9A). PERMANOVA confirmed that there was no difference in community composition (PERMANOVA, n = 16, p > 0.05) between untreated samples and those from samples treated with ethanol. Further, hierarchal clustering demonstrated that communities from both treated and untreated samples were intermixed samples collected from the same animals tended clustered to be within a single large clade (Fig 9B). However, only six of the 16 ethanol stabilized samples were highly similar to untreated samples collected from the same animals (Fig 9B). The major driver of clade formation was inter-sample differences between predominant taxa (Fig 9B). For example, communities dominated by Mycoplasmataceae formed the largest clade, while those with higher relative abundance of Pasteurellaceae, Moraxellaceae, or Prevotellaceae were in different clades (Fig 9B). Shifts in the relative abundance of ASVs were small between untreated and ethanol stabilized communities (average r = 0.902), albeit with a very large range (r = 0.408 and r = 0.995). The majority of samples exhibited shifts similar to those seen in ethanol stabilized fecal communities compared to fresh fecal communities (Figs 5–7B).

## Discussion

This study utilized 16S rRNA gene sequencing to investigate the effects of two stabilization solutions, and temperature, over time in the absence of biological variation on maintaining microbial community structure in bovine fecal and nasopharyngeal swab samples. Sample collection in remote areas or under time constraints may not provide an opportunity to immediately cool and freeze samples in ultracold freezers and researchers may need to utilize a preservation method to help maintain representative microbial community structures. Further, the number of studies targeting microbial communities within the animal and agricultural sciences is growing rapidly, and along with an ever-increasing need for larger sample sizes, temporal scales, and environmental coverage the availability and effectiveness of stabilization methods is of critical importance for researchers.

In the absence of biological variation, both stabilization solutions introduced immediate bias into the microbial community structure of bovine fecal samples, though stabilization with OMNIgene^®^•GUT solution performed considerably better than stabilization with 100% ethanol. Some previous studies based on human fecal samples have recommended stabilization with ethanol for sample storage [35,48], while another concluded it affected community composition [32]. The results here suggest that stabilization with ethanol is not ideal under most common storage situations. The immediate and short-term bias introduced by ethanol appears to result in altered relative abundances of variety of taxa within the classes Clostridia and Bacteroidia. These represent two of the most abundant classes found within the bovine GIT across populations from different geographic locations and environmental conditions [7,49,50], suggesting that stabilization with ethanol is likely not optimal for the majority of bovine GIT sample storage. Additionally, variation among technical replicates was much higher in samples stabilized with ethanol versus untreated samples or those stabilized in OMNIgene^®^•GUT solution. While stabilization with OMNIgene^®^•GUT also introduced immediate bias, it was considerably smaller and was limited to rare taxa.

After 7-days and 14-days storage, stabilization with OMNIgene preserved original community structure in bovine feces better than flash-freezing or ethanol stabilization, particularly at temperatures above freezing. Below freezing, differences between untreated and stabilized communities were minimal and generally limited to less prevalent taxa, suggesting that use of a stabilization solution may not be necessary when using short-term storage in an ultracold freezer, unless study objectives require rare biosphere characterization. When it is not possible to immediately freeze samples, storage conditions were associated with dramatic impacts on microbial community compositions. Microbial communities shifted substantially over time without use of preservation, which was expected as it is has been known for over a century that specific taxa will outperform others (i.e., bottle effect) given the different nutrient conditions within the sample storage tube [51–53]. Preservation of microbial community structure in feces stabilized with ethanol stored above freezing was considerably better than untreated communities, but OMNIgene^®^•GUT stabilization resulted in much better-preserved communities than both ethanol and untreated communities. Previous work corroborates our findings that OMNIgene^®^•GUT performs better than other stabilization solutions [35,54]. Indeed, stabilization with OMNIgene^®^•GUT for short-term storage (< 14 days) at 4°C and even at room temperature, resulted in a considerably better-preserved microbial community structures in bovine fecal samples.

Stabilization with ethanol had a smaller impact on bovine respiratory microbial communities than did inter-individual variation when stored at -80°C and shipped from one laboratory to another on ice. In fecal samples, stabilization with ethanol has been demonstrated to introduce differences equal or less than inter-individual variation [35], but to our knowledge these results are the first to compare stabilization of respiratory communities collected with swabs. However, further work is needed to compare flash-frozen and ethanol stabilized samples with fresh samples representative of the original community structure. Additionally, we acknowledge that for both our fecal and respiratory communities, a larger series of samples with differing composition may have identified differences not observed here.

## Supporting information

S1 Table Proportion of ASVs classified at the taxonomic ranks of phylum, class, order, family, and genus for fecal and respiratory samples. (DOCX)

S2 Table Metrics regarding the concentration of DNA isolated from bovine feces stored without stabilization solution, ethanol, or OMNIgene GUT. Metrics are reported for samples isolated immediately after collection, after 7-days storage, and 14-days storage.
(DOCX)

S3 Table PERMANOVA and PERMDISP results from comparisons between fecal microbial communities isolated immediately after collection and stored in either no stabilization solution, ethanol, or OMNIGene GUT based on generalized UniFrac distances. Significant values are bolded (P < 0.05).
(DOCX)

S4 Table Metrics regarding the concentration of DNA isolated from respiratory swabs stored without stabilization solution or ethanol. (DOCX)
