# Supplementary material for: Effectiveness of stabilization methods for the immediate and short-term preservation of bovine fecal and upper respiratory tract genomic DNA
Source: PLoS One. 2024 Apr 2;19(4):e0300285. doi: 10.1371/journal.pone.0300285 (PMC10987004; doi:10.1371/journal.pone.0300285)
Supplement: S1 Table — (DOCX) [file pone.0300285.s001.docx]

**Table S1.** Proportion of ASVs classified at the taxonomic ranks of phylum, class, order, family, and genus for fecal and respiratory samples.

|  | **Phylum** | **Class** | **Order** | **Family** | **Genus** |
| --- | --- | --- | --- | --- | --- |
| **Fecal** | 99.99% | 99.95% | 99.35% | 99.22% | 93.68% |
| **Respiratory** | 99.56% | 99.56% | 99.55% | 99.28% | 95.38% |
