# Supplementary material for: Effectiveness of stabilization methods for the immediate and short-term preservation of bovine fecal and upper respiratory tract genomic DNA
Source: PLoS One. 2024 Apr 2;19(4):e0300285. doi: 10.1371/journal.pone.0300285 (PMC10987004; doi:10.1371/journal.pone.0300285)
Supplement: S2 Table — Metrics are reported for samples isolated immediately after collection, after 7-days storage, and 14-days storage. (DOCX) [file pone.0300285.s002.docx]

**Table S2**. Metrics regarding the concentration of DNA isolated from bovine feces stored without stabilization solution, ethanol, or OMNIgene GUT. Metrics are reported for samples isolated immediately after collection, after 7-days storage, and 14-days storage.

| **FRESH** | **Minimum (ng/μL)** | **Maximum (ng/μL)** | **Mean (ng/μL) ± SEM** |
| --- | --- | --- | --- |
| No treatment | 46.40 | 68.00 | 59.30 ± 4.68 |
| OMNIgene GUT | 33.80 | 47.90 | 40.45 ± 3.62 |
| Ethanol | 10.08 | 15.79 | 13.54 ± 1.39 |

| **7-DAYS** | **Minimum (ng/μL)** | **Maximum (ng/μL)** | | **Mean (ng/μL) ± SEM** |
| --- | --- | --- | --- | --- |
| **-80°C** |  | |  |  |
| No treatment | 46.30 | | 57.80 | 52.53 ± 2.36 |
| OMNIgene GUT | 26.90 | | 47.10 | 41.00 ± 1.67 |
| Ethanol | 16.81 | | 24.70 | 21.15 ± 4.77 |
| **-20°C** |  | |  |  |
| No treatment | 53.50 | | 65.70 | 62.15 ± 2.91 |
| OMNIgene GUT | 28.70 | | 42.80 | 35.70 ± 2.24 |
| Ethanol | 12.90 | | 23.10 | 19.38 ± 2.88 |
| **+4°C** |  | |  |  |
| No treatment | 55.20 | | 73.80 | 64.85 ± 3.87 |
| OMNIgene GUT | 31.00 | | 39.30 | 35.38 ± 2.03 |
| Ethanol | 7.06 | | 16.67 | 12.42 ± 1.76 |
| **+20°C** |  | |  |  |
| No treatment | 27.70 | | 72.10 | 52.30 ± 10.47 |
| OMNIgene GUT | 42.20 | | 50.10 | 46.68 ± 1.98 |
| Ethanol | 1.05 | | 11.39 | 5.51 ± 2.16 |

| **14-DAYS** | **Minimum (ng/μL)** | **Maximum (ng/μL)** | | **Mean (ng/μL) ± SEM** |
| --- | --- | --- | --- | --- |
| **-80°C** |  | |  |  |
| No treatment | 61.40 | | 79.40 | 70.80 ± 3.70 |
| OMNIgene GUT | 35.10 | | 44.10 | 39.13 ± 1.92 |
| Ethanol | 17.05 | | 19.61 | 18.84 ± 0.60 |
| **-20°C** |  | |  |  |
| No treatment | 68.20 | | 79.30 | 73.33 ± 2.59 |
| OMNIgene GUT | 33.60 | | 43.60 | 39.93 ± 2.17 |
| Ethanol | 11.28 | | 32.30 | 19.48 ± 4.51 |
| **+4°C** |  | |  |  |
| No treatment | 36.70 | | 92.50 | 68.38 ± 13.38 |
| OMNIgene GUT | 35.10 | | 39.90 | 37.49 ± 0.98 |
| Ethanol | 15.88 | | 23.50 | 18.14 ± 1.79 |
| **+20°C** |  | |  |  |
| No treatment | 61.40 | | 74.30 | 69.05 ± 2.74 |
| OMNIgene GUT | 34.30 | | 40.70 | 37.83 ± 1.39 |
| Ethanol | 4.22 | | 5.76 | 4.71 ± 0.36 |
