# Supplementary material for: Effectiveness of stabilization methods for the immediate and short-term preservation of bovine fecal and upper respiratory tract genomic DNA
Source: PLoS One. 2024 Apr 2;19(4):e0300285. doi: 10.1371/journal.pone.0300285 (PMC10987004; doi:10.1371/journal.pone.0300285)
Supplement: S3 Table — Significant values are bolded (P < 0.05). (DOCX) [file pone.0300285.s003.docx]

**Table S3**. PERMANOVA and PERMDISP results from comparisons between fecal microbial communities isolated immediately after collection and stored in either no stabilization solution, ethanol, or OMNIGene GUT based on generalized UniFrac distances. Significant values are bolded (P < 0.05).

|  | **Df** | **SS** | **Pseudo-F** | **R^2^** | **p-adj.** | **PERMDISP (p-adj.)** |
| --- | --- | --- | --- | --- | --- | --- |
| No Treatment vs. EtOH | 1 | 0.068 | 44.009 | 0.880 | **0.041** | **0.012** |
| No Treatment vs. OMNIGene | 1 | 0.006 | 5.832 | 0.492 | **0.027** | 0.154 |
| EtOH vs. OMNIGENE | 1 | 0.059 | 36.931 | 0.860 | **0.026** | 0.056 |

Abbreviations: Df, degrees of freedom; SS, sum of squares
