# Supplementary material for: Effectiveness of stabilization methods for the immediate and short-term preservation of bovine fecal and upper respiratory tract genomic DNA
Source: PLoS One. 2024 Apr 2;19(4):e0300285. doi: 10.1371/journal.pone.0300285 (PMC10987004; doi:10.1371/journal.pone.0300285)
Supplement: S4 Table — (DOCX) [file pone.0300285.s004.docx]

**Table S4**. Metrics regarding the concentration of DNA isolated from respiratory swabs stored without stabilization solution or ethanol.

|  | **Minimum (ng/μL)** | **Maximum (ng/μL)** | **Mean (ng/μL) ± SEM** |
| --- | --- | --- | --- |
| **No treatment** | 51.50 | 134.50 | 88.65 ± 6.30 |
| **Ethanol** | 38.40 | 108.00 | 76.76 ± 4.84 |
